# Sensitivity of the Point-of-Care Circulating Cathodic Antigen Urine Cassette Test for Diagnosis of *Schistosoma mansoni* in Low-Endemicity Settings in Côte d’Ivoire

**DOI:** 10.4269/ajtmh.18-0550

**Published:** 2018-10-01

**Authors:** Rufin K. Assaré, Mathieu B. I. Tra, Mamadou Ouattara, Eveline Hürlimann, Jean T. Coulibaly, Eliézer K. N’Goran, Jürg Utzinger

**Affiliations:** 1 Swiss Tropical and Public Health Institute, Basel, Switzerland;; 2 University of Basel, Basel, Switzerland;; 3 Unité de Formation et de Recherche Biosciences, Université Félix Houphouët-Boigny, Abidjan, Côte d'Ivoire;; 4 Centre Suisse de Recherches Scientifiques en Côte d’Ivoire, Abidjan, Côte d’Ivoire

## Abstract

The sensitivity of a point-of-care circulating cathodic antigen (POC-CCA) urine cassette test for diagnosis of *Schistosoma mansoni* in low-endemicity settings is poorly understood. We conducted a cross-sectional survey in 14 villages in western Côte d’Ivoire and diagnosed children aged 9–12 years for schistosomiasis. Two stool samples were subjected to triplicate Kato–Katz thick smears each for diagnosis of *S. mansoni*, whereas a single urine sample was examined by POC-CCA for *S. mansoni,* filtration for *Schistosoma haematobium,* and reagent strip for microhematuria. According to the Kato–Katz technique, we found 45 out of 681 children positive for *S. mansoni* (6.6%) with a mean intensity among infected children of 72.2 eggs per gram of stool. Point-of-care circulating cathodic antigen revealed a prevalence of *S. mansoni* of 33.0% when trace results were considered positive and 12.5% when trace results were considered negative. Eggs of *S. haematobium* were found in eight participants (1.2%), whereas the prevalence of microhematuria was 13.5%. A single POC-CCA urine cassette test revealed a several-fold higher prevalence of *S. mansoni* than multiple Kato–Katz thick smears in this low-endemicity area. Our findings have important ramifications for choosing an appropriate diagnostic tool in low-endemic areas that might be targeted for elimination.

## INTRODUCTION

Schistosomiasis mansoni is endemic in the western part of Côte d’Ivoire.^1–4^ Facilitated by the Schistosomiasis Consortium for Operational Research and Evaluation (SCORE), a 5-year cluster-randomized trial was launched in 2011 in 75 villages of western Côte d’Ivoire.^5^ Three school-based preventive chemotherapy schedules were implemented, which considerably reduced the prevalence and intensity of *Schistosoma mansoni* infection already in the first year of intervention.^5,6^ Toward the end of the trial, school children were invited to submit three stool samples over consecutive days. Stool samples were subjected to the Kato–Katz technique that is widely used in epidemiologic investigations pertaining to intestinal schistosomiasis.^5–7^

Although the Kato–Katz technique is a valuable tool at the onset of large-scale control programs, it is less useful for disease surveillance in settings where the prevalence and intensity decreased after several rounds of praziquantel treatment.^7,8^ Indeed, the Kato–Katz method underestimates the true prevalence in low-endemicity settings and shows low sensitivity after praziquantel treatment.^8–11^ This urges for novel and more sensitive diagnostic approaches to monitor the prevalence and intensity of *S. mansoni* in areas with established control programs that implement preventive chemotherapy. The aforementioned SCORE trial provided an opportunity to assess the sensitivity of a point-of-care circulating cathodic antigen (POC-CCA) urine cassette test for the diagnosis of *S. mansoni* in the final year of treatment intervention when the endemicity of schistosomiasis mansoni was suspected to be low.

## MATERIALS AND METHODS

### Ethics.

Ethics approval was obtained from the “Comité National d’Ethique et de la Recherche” of the Ministry of Health in Côte d’Ivoire (reference no. 046/MSHP/CNER-kp; date of assignment: May 30, 2016). Local health and education authorities and village leaders were informed about the purpose and procedures of the study. Participation was voluntary, and hence, children could withdraw any time without further obligation. At the end of the study, all children and nonpregnant girls aged between 5 and 15 years were given a single 40 mg/kg oral dose of praziquantel, free of charge, following World Health Organization guidelines.^12^

### Study design.

The main outcome of the study was the prevalence of *S. mansoni*, as assessed by the standard Kato–Katz technique using stool samples, to be compared with the *S. mansoni* prevalence based on the POC-CCA test using a single urine sample. The study was conducted in October 2016 in three regions of western Côte d’Ivoire (geographic coordinates: lat. 06°32′42.0″–07°36′54.8″ N and long. 06°44′09.8″–07°33′48.9″ W). It followed a cross-sectional design and was carried out in 14 schools purposely selected based on low prevalence (< 10%) of *S. mansoni* infection among 9- to 12-year-old children, as detected by the Kato–Katz method^13^ in the final survey of the SCORE study after two or four treatments with praziquantel over a 4-year period.^4,14^ Among the selected villages, Ziondrou and Gbadrou were persistent hot spots as defined according to changes in the prevalence of *S. mansoni* from year 1 to year 2 with slight increases of 15.6% and 10.5% after the first round of praziquantel treatment, respectively.^15^ These two villages were selected for assessing the main features that contribute to the increase of *S. mansoni* prevalence. In the remaining 12 villages, the prevalence of *S. mansoni* ranged from nil to 10%. All children aged 9–12 years in the 14 study villages who attended school, had no chronic disease conditions, and were nonpregnant were eligible to participate.

### Stool and urine collection.

In each school, 50 children who met the eligibility criteria were randomly selected. Written informed consent was obtained from parents or guardians, whereas children provided oral assent. Children were given two pre-labeled plastic containers and were invited on the next day to provide a portion of their fresh morning stool in one container and a urine sample produced between 10:00 and 12:00 hours in the second container. For each child, two stool samples were collected on consecutive days, whereas only a single urine sample was collected on the first day.

Triplicate 41.7 mg Kato–Katz thick smears were prepared from each stool sample and examined under a microscope using a standard protocol.^13^ The number of *S. mansoni* eggs was counted and recorded for each thick smear. Mean egg counts were multiplied by a factor of 24 to obtain a measure of infection intensity, as expressed as the number of eggs per gram of stool (EPG). Children harboring *S. mansoni* eggs were categorized into 1) light (1–99 EPG), 2) moderate (100–399 EPG), and 3) heavy (≥ 400 EPG) intensity of infection.^16^ In addition, eggs of hookworm, *Ascaris lumbricoides*, *Enterobius vermicularis*, *Hymenolepis* spp., *Taenia* spp., and *Trichuris trichiura* were investigated, but will not be presented in this article.

Urine samples were subjected to a filtration method, counting the number of *Schistosoma haematobium* eggs in 10 mL of urine, visual inspection for macrohematuria, and reagent strips (Hemastix; Siemens Healthcare Diagnostics GmbH, Zurich, Switzerland; batch number: ref 2816A) for microhematuria.^17^ The intensity of *S. haematobium* was classified as light (1–49 eggs/10 mL of urine) or heavy (≥ 50 eggs/10 mL of urine).^16^ *Schistosoma haematobium* data were considered in the present research to evaluate the eventual cross-reactivity in urinary tract infection. The results of the reagent strips were stratified into negative and positive, the latter including non-hemolyzed and hemolyzed trace of blood, 1+, 2+, and 3+, according to the manufacturer’s instructions. In addition, a POC-CCA (Rapid Medical Diagnostics, Pretoria, South Africa; batch number: 50182) was performed.^18^ In brief, one drop of urine was added in the well of the cassette. Once completely absorbed, one drop of test buffer was added to the well and allowed to develop for 20 minutes. The tests were read by an experienced laboratory technician and scored as negative, trace, or positive.

### Statistical analysis.

Data were entered into Microsoft Excel 2010 (Microsoft Corporation, Redmond, WA). Statistical analyses were performed with STATA version IC13.1 (Stata Corporation, College Station, TX). A χ^2^ test was conducted and 95% confidence intervals (CIs) were calculated, considering statistical significance for *P* values < 0.05 and non-overlapping 95% CIs. We considered results from the Kato–Katz technique as the diagnostic “gold” standard. Prevalence of *S. mansoni* using three different diagnostic interpretations (i.e., multiple Kato–Katz thick smears, POC-CCA with trace considered positive and POC-CCA with trace considered negative), *S. haematobium,* and microhematuria were displayed by geographical location using Arc Map 10.5 (Environmental Systems Research Institute, Inc., Redlands, CA).

## RESULTS

Overall, 681 children aged 9–12 years were included in the final analyses; 308 (45.2%) females and 373 (54.8%) males. Most of the children provided two stool samples (*N* = 641, 94.1%), whereas the remaining 40 children had a single stool sample examined. Microscopic examination of stool and urine samples revealed that 53 children (7.8%) harbored eggs of *S. mansoni* and *S. haematobium*. No *S. mansoni*–*S. haematobium* coinfection was observed.

Eggs of *S. mansoni* (based on six Kato–Katz thick smears for 641 children and three Kato–Katz thick smears for the remaining children) were found in 45 children, owing to an overall prevalence of 6.6% (95% CI: 4.7–8.4%). Males had a slightly higher *S. mansoni* prevalence than females (6.9% *versus* 6.1%). At the unit of the school, the prevalence of *S. mansoni* ranged from nil to 22.0% (Table 1). Twelve schools had a prevalence of *S. mansoni* below 10%, whereas the remaining two schools were above this threshold (Gbadrou and Ziondrou). The arithmetic mean egg count among *S. mansoni*–positive individuals was 72.2 EPG, ranging from 4.0 (Siambly) to 234.7 EPG (Gbadrou) (Table 2). Most of the infected children (86.7%) had low-intensity *S. mansoni* infections with less than 100 EPG.

**Table 1:** Prevalence of *Schistosoma mansoni* and *Schistosoma haematobium* infections in 9- to 12-year-old children from 14 schools in western Côte d’Ivoire, in a cross-sectional survey carried out in October 2016

Village	No. of children examined	*S. mansoni*				Hematuria		*S. haematobium*	
		Point-of-care circulating cathodic antigen urine cassette test*		Kato–Katz method		Reagent strip		Urine filtration	
		No. (%) of children positive	95% CI	No. (%) of children positive	(95% CI)	No. (%) of children with micro-hematuria	(95% CI)	No. (%) of children positive	(95% CI)
Baoulé Carrefour	50	15 (30.0)	(16.8–43.2)	2 (4.0)	(−1.6–9.6)	4 (8.0)	(0.2–15.8)	0 (0.0)	–
Gbadrou	50	22 (44.0)	(29.7–58.8)	6 (12.0)	(2.7–21.3)	13 (26.0)	(13.4–38.6)	0 (0.0)	–
Gregbeu	50	12 (24.0)	(11.7–36.2)	0 (0.0)	–	0 (0.0)	–	0 (0.0)	–
Klangbolably	49	16 (32.7)	(19.0–46.3)	3 (6.0)	(−0.8–13.1)	13 (26.5)	(13.7–39.3)	0 (0.0)	–
Koua	50	14 (28.0)	(15.1–40.8)	4 (8.0)	(0.2–15.8)	6 (12.0)	(2.7–21.3)	0 (0.0)	–
Mahinahi	47	16 (34.0)	(19.9–48.1)	1 (2.1)	(−2.1–6.4)	3 (6.4)	(−0.8–13.6)	0 (0.0)	–
Mona	49	18 (36.7)	(22.7–50.7)	2 (4.1)	(−1.6–9.8)	8 (16.3)	(5.6–27.1)	0 (0.0)	–
Semien	36	7 (19.4)	(5.8–33.0)	3 (6.3)	(−1.2–17.8)	0 (0.0)	–	0 (0.0)	–
Siambly	50	12 (24.0)	(11.7–36.2)	1 (2.0)	(−2.0–6.0)	0 (0.0)	–	0 (0.0)	–
Tobly Bangolo	50	11 (22.0)	(10.1–33.9)	4 (8.0)	(0.2–15.8)	5 (10.0)	(1.4–18.6)	0 (0.0)	–
Ziondrou	50	25 (50.0)	(35.6–64.5)	11 (22.0)	(10.1–33.9)	9 (18.0)	(7.0–29.0)	2 (4.0)	(−1.6–9.6)
Zouatta 2	50	22 (44.0)	(29.7–58.2)	3 (6.0)	(−0.8–12.8)	22 (44.0)	(29.7–58.3)	1 (2.0)	(−2.0–6.0)
Zoukougbeu	50	22 (44.0)	(29.7–58.2)	3 (6.0)	(−0.8–12.8)	8 (16.0)	(5.5–26.5)	4 (8.0)	(0.2–15.8)
Zê	50	13 (26.0)	(13.4–38.6)	2 (4.0)	(−1.6–9.6)	1 (2.0)	(−2.0–6.0)	1 (2.0)	(−2.0–6.0)
Total	681	225 (33.0)	(29.5–36.6)	45 (6.6)	(4.7–8.4)	92 (13.5)	(10.9–16.1)	8 (1.2)	(0.3–1.9)

* Trace results considered as positive.

**Table 2:** Intensity of *Schistosoma mansoni* and *Schistosoma haematobium* infections in 9- to 12-year-old children from 14 schools in western Côte d’Ivoire, in a cross-sectional survey carried out in October 2016

Village	*S. mansoni*		*S. haematobium*	
	AM (EPG)	(95% CI)	Eggs/10 mL	(95% CI)
Baoulé Carrefour	12.0	(−38.8–62.8)	0.0	–
Gbadrou	234.7	(−325.8–795.2)	0.0	–
Gregbeu	0.0	–	0.0	–
Klangbolably	21.3	(−1.6–44.3)	0.0	–
Koua	13.0	(5.0–21.0)	0.0	–
Mahinahi	88.0	–	0.0	–
Mona	56.0	(−503.1–615.1)	0.0	–
Semien	60.0	(−52.0–172.0)	0.0	–
Siambly	4.0	–	0.0	–
Tobly Bangolo	10.0	(−2.2–22.2)	0.0	–
Ziondrou	76.4	(0.3–152.4)	6.5	(−50.7−63.7)
Zouatta 2	72.0	(−186.4–330.3)	11.0	–
Zoukougbeu	49.3	(−137.2–235.8)	21.8	(−5.3–48.8)
Zê	36.0	(−167.3–239.3)	37.0	–
Total	72.2	(11.3–133.1)	18.5	(5.7–31.3)

AM (EPG) = arithmetic mean egg count (eggs per gram).

**Figure 1.**
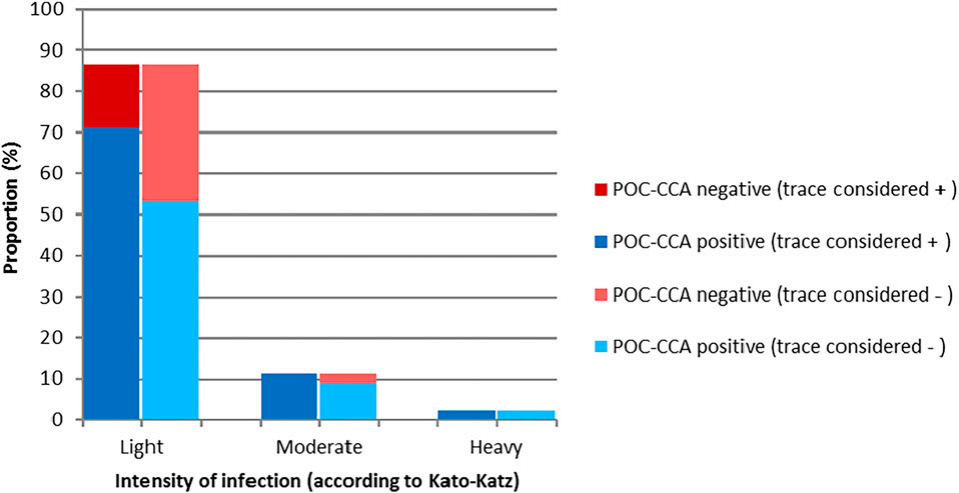
*Schistosoma mansoni* infection by intensity category and point-of-care circulating cathodic antigen (POC-CCA) performance in children found positive by the Kato–Katz technique (*N* = 45).

A single POC-CCA urine cassette test revealed *S. mansoni* prevalence of 33.0% (225 positive children; 95% CI: 29.5–36.6%) when considering trace results as positive. At the unit of the school, the *S. mansoni* prevalence using POC-CCA ranged from 19.4% (Semien) to 50.0% (Ziondrou). Considering trace results as negative, the respective prevalence of *S. mansoni* was 12.5% (85 positive children; 95% CI: 9.9–14.9%). Within the Kato–Katz positives, 29 of 45 children (64.4%) were confirmed by POC-CCA testing considering trace as negative and 38 children (84.4%) considering trace as positive. In the seven Kato–Katz–positive children who showed negative POC-CCA results (if trace included as positive), the mean egg counts were consistently below 100 EPG. Hence, if trace results were included as positive, the POC-CCA only missed light-intensity infections with an overall false-negative proportion of 15.5% (Figure 1). If considering trace results as negative, the sensitivity and specificity of the POC-CCA urine cassette test for *S. mansoni* diagnosis was 64.4% and 91.2%, respectively. The corresponding sensitivity and specificity if trace results were considered positive were 84.4% and 70.6%, respectively.

No macrohematuria was observed. Reagent strip tests found 92 (13.5%) children with microhematuria. The *S. haematobium* prevalence, according to a single urine filtration, was very low (1.2%). Eggs of *S. haematobium* were found in the urine of children from only four schools, whereas microhematuria was observed in 11 schools. The arithmetic mean egg count of *S. haematobium* was 18.5 eggs/10 mL (95% CI: 5.7–31.3 eggs/10 mL) ranging from zero to 37.0 eggs/10 mL (Table 2). All *S. haematobium*–positive children had light-intensity infections.

**Figure 2.**
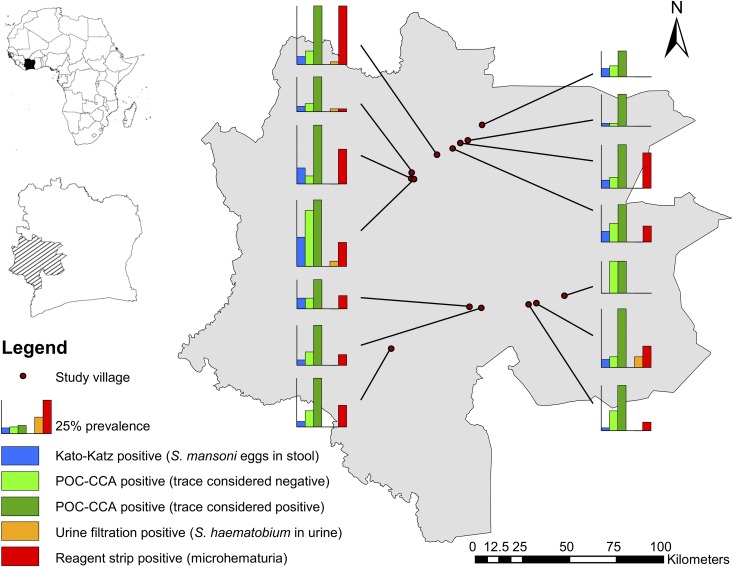
Map of the study area in the western part of Côte d’Ivoire showing the distribution of *Schistosoma mansoni* (based on Kato–Katz and point-of-care circulating cathodic antigen [POC-CCA]) and *Schistosoma haematobium* infections and the percentage of microhematuria positives in each of 14 schools examined in October 2016.

Figure 2 displays the spatial distribution of schistosomiasis in the study area, according to Kato–Katz and POC-CCA for *S. mansoni*, and urine filtration for *S. haematobium* and microhematuria using reagent strips.

## DISCUSSION

A preceding multi-country SCORE study revealed that a single POC-CCA urine cassette test is at least as sensitive as triplicate Kato–Katz thick smears for the diagnosis of *S. mansoni*.^19^ However, relatively little is known about the sensitivity of POC-CCA in low-endemicity settings, which are gaining in importance because of large-scale preventive chemotherapy programs.^20^ Indeed, in the present study, the baseline prevalence of *S. mansoni* at the onset of a 5-year cluster-randomized trial in 2011 in the 14 villages studied here was 19.0%,^5^ whereas 5 years later after two or four rounds of praziquantel administration, it had dropped to 6.6% (current data). These estimates are based on the most widely used diagnostic approach for *S. mansoni*—the Kato–Katz method.^7^ According to the POC-CCA urine cassette test with trace results considered positive, we found a 5-fold higher prevalence of *S. mansoni* (33.0%), whereas POC-CCA with trace results considered negative resulted in a 2-fold higher prevalence (12.5%) compared with stool microscopy. The POC-CCA has been proposed for the mapping of *S. mansoni*.^19^ Recent studies among African migrants in Europe conjectured that the POC-CCA urine cassette test might also be considered for individual diagnosis.^21–23^

Our findings indicate that the effect of regular deworming with praziquantel is overestimated, at least when one is using prevalence reduction based on an insensitive diagnostic method, such as the Kato–Katz technique. Our results might thus raise concern about the global strategy of schistosomiasis control, and call for more sensitive diagnostic tools, as the focus shifts from morbidity control to interrupting transmission. The POC-CCA appears to be a useful tool in low-endemicity settings.^24^ Our results corroborate with the findings from a recent article, analyzing data of 19 studies by comparing *S. mansoni* prevalence revealed by Kato–Katz and POC-CCA. Indeed, POC-CCA urine cassette test results found *S. mansoni* prevalence estimates that were between 1.5-fold and up to 6-fold higher than standard Kato–Katz.^25^ Cross-reactivity from urinary tract infection (in school-aged children in our setting most likely due to *S. haematobium* infection) and hematuria cannot be ruled out completely. However, we would like to point out that in two study villages (i.e., Gregbeu and Siambly), no or only one of the participating children excreted *S. mansoni* eggs, as detected by Kato–Katz. In contrast, a single POC-CCA revealed an *S. mansoni* prevalence of 24.0%. In these villages, no *S. haematobium* was found (neither by urine filtration nor by reagent strip), and hence, we assume the risk of potential cross-reactivity as minimal in our study setting. Higher sensitivity of the POC-CCA may also be explained by taking into account findings from animal models. These showed detectable CCA levels for *S. mansoni* as early as 3 weeks after experimental infection, highlighting the potential to also capture infection status with juvenile stages not yet producing eggs.^26^ We can further exclude discrepancies between egg excretion and remaining antigen from recently killed adult worms, because there was more than a 15-month span between the last treatment round and the current cross-sectional survey. It should also be noted that standard urine filtration and microscopy revealed a very low prevalence of *S. haematobium* (1.2%), whereas reagent strip testing revealed a prevalence of microhematuria of 13.5%. This observation corroborates findings from previous studies in Côte d’Ivoire and Chad, where a “background” prevalence of microhematuria of about 13% was observed in the absence of *S. haematobium* eggs in children’s urine.^27^ This result may be due to menstrual blood loss among the oldest females included in our study (e.g., aged 12 years), as this can lead to reagent strip–positive results. Yet, it should also be noted that examination of larger volumes of urine (e.g., 50 mL and more) and duplication urine might have revealed considerably higher *S. haematobium* prevalence rates.

Previous studies assessing the sensitivity of POC-CCA for *S. mansoni* diagnosis elsewhere in Côte d’Ivoire and in Cameroon showed that *S. haematobium* and soil-transmitted helminth infections did not influence POC-CCA results.^18,28^ The map in Figure 2 exemplifies how focally schistosomiasis is distributed. For example, in the three schools situated in a perimeter of less than 5 km in the northwestern part of the study area (i.e., Gbadrou, Zê, and Ziondrou), *S. mansoni* and microhematuria showed considerable spatial heterogeneity.

A limitation of our study is that, unlike other studies,^10,11,29^ we did not further stratify POC-CCA–positive results using band strengths. Our data, thus, did not allow for direct correlation of egg count and rapid test-based intensity measures. We did, however, observe that false-negative POC-CCA results were predominantly found in individuals with egg counts below 100 EPG and that they depended on the interpretation of the trace results to a similar extent as in earlier studies.^29^

## CONCLUSION

Our results confirm that a single POC-CCA urine cassette test is considerably more sensitive than multiple Kato–Katz thick smears in a low-endemicity setting of schistosomiasis mansoni after multiple rounds of preventive chemotherapy. Point-of-care circulating cathodic antigen might, therefore, be considered as a tool for *S. mansoni* rapid risk profiling. In view of the findings presented here, the potential role of a POC-CCA urine cassette test for determining the impact of preventive chemotherapy with praziquantel, particularly in low-endemicity settings, should be investigated.
